# Recent advances and perspectives of metabolomics-based investigations in Parkinson’s disease

**DOI:** 10.1186/s13024-018-0304-2

**Published:** 2019-01-11

**Authors:** Yaping Shao, Weidong Le

**Affiliations:** 1 Center for Clinical Research on Neurological Diseases, The First Affiliated Hospital, Dalian Medical University, Dalian, China; 2 Liaoning Provincial Key Laboratory for Research on the Pathogenic Mechanisms of Neurological Diseases, The First Affiliated Hospital, Dalian Medical University, Dalian, China

**Keywords:** Parkinson’s disease, Metabolomics, Biomarker, Metabolic pathway

## Abstract

Parkinson’s disease (PD) is the second most prevalent neurodegenerative disease of the central nervous system (CNS), which affects mostly older adults. In recent years, the incidence of PD has been dramatically increasing with the aging population expanding. Due to the lack of effective biomarkers, the accurate diagnosis and precise treatment of PD are currently compromised. Notably, metabolites have been considered as the most direct reflection of the physiological and pathological conditions in individuals and represent attractive candidates to provide deep insights into disease phenotypes. By profiling the metabolites in biofluids (cerebrospinal fluid, blood, urine), feces and brain tissues, metabolomics has become a powerful and promising tool to identify novel biomarkers and provide valuable insights into the etiopathogenesis of neurological diseases. In this review, we will summarize the recent advancements of major analytical platforms implemented in metabolomics studies, dedicated to the improvement and extension of metabolome coverage for in-depth biological research. Based on the current metabolomics studies in both clinical populations and experimental PD models, this review will present new findings in metabolomics biomarkers research and abnormal metabolic pathways in PD, and will discuss the correlation between metabolomic changes and clinical conditions of PD. A better understanding of the biological underpinning of PD pathogenesis might offer novel diagnostic, prognostic, and therapeutic approaches to this devastating disease.

## Background

Parkinson’s disease (PD) is a progressive, multi-focal neurodegenerative disorder, affecting approximately 1% of people over the age of 60 [1, 2]. The diagnosis of PD mainly relies on clinical symptoms, medical history, and response to dopaminergic treatment, which results in a high rate of misdiagnosis in the clinical practice of PD [3, 4]. In addition, the clinical manifestations of PD patients usually lag behind the underlying pathological changes in the brain, making the early diagnosis of PD a great challenge [5]. Currently, the most commonly used therapeutic strategy for PD, dopamine replacement therapy, can only improve the clinical motor symptoms and is incapable of slowing or halting disease progression. Markedly, long-term medical treatment can lead to serious, irreversible motor complications, such as L-dopa induced dyskinesia (LID) [6]. Although, a range of biomarkers derived from clinical, neuroimaging, genetic, and biochemical studies have been proposed [7–12], sensitive, specific, and reliable biomarkers for PD remain elusive. Deterioration of dopaminergic neurons within the substantia nigra pars compacta and the accumulation of intracytoplasmic inclusions known as Lewy Bodies are hallmarks of the PD pathobiology [13]. Currently, the proposed hypotheses for the pathogenesis of PD include protein misfolding and aggregation, mitochondrial injury, oxidative stress and inflammation [14, 15]. However, since PD is a multifactorial disease, it is likely that multiple mechanisms may contribute to its pathogenesis. Despite decades of research, the underlying etiopathogenesis of PD is still not fully elucidated. Given the lack of knowledge regarding the mechanisms that regulate the onset and progression of the disease pathology, new approaches dedicated to the discovery of specific biomarkers that offer more accurate diagnosis, and better monitoring of PD progression and prognosis are in urgent need. Furthermore, the identification of reliable targets might lead to the development of novel drugs, which could reverse the neurodegeneration and progression of PD.

**Fig. 1:**
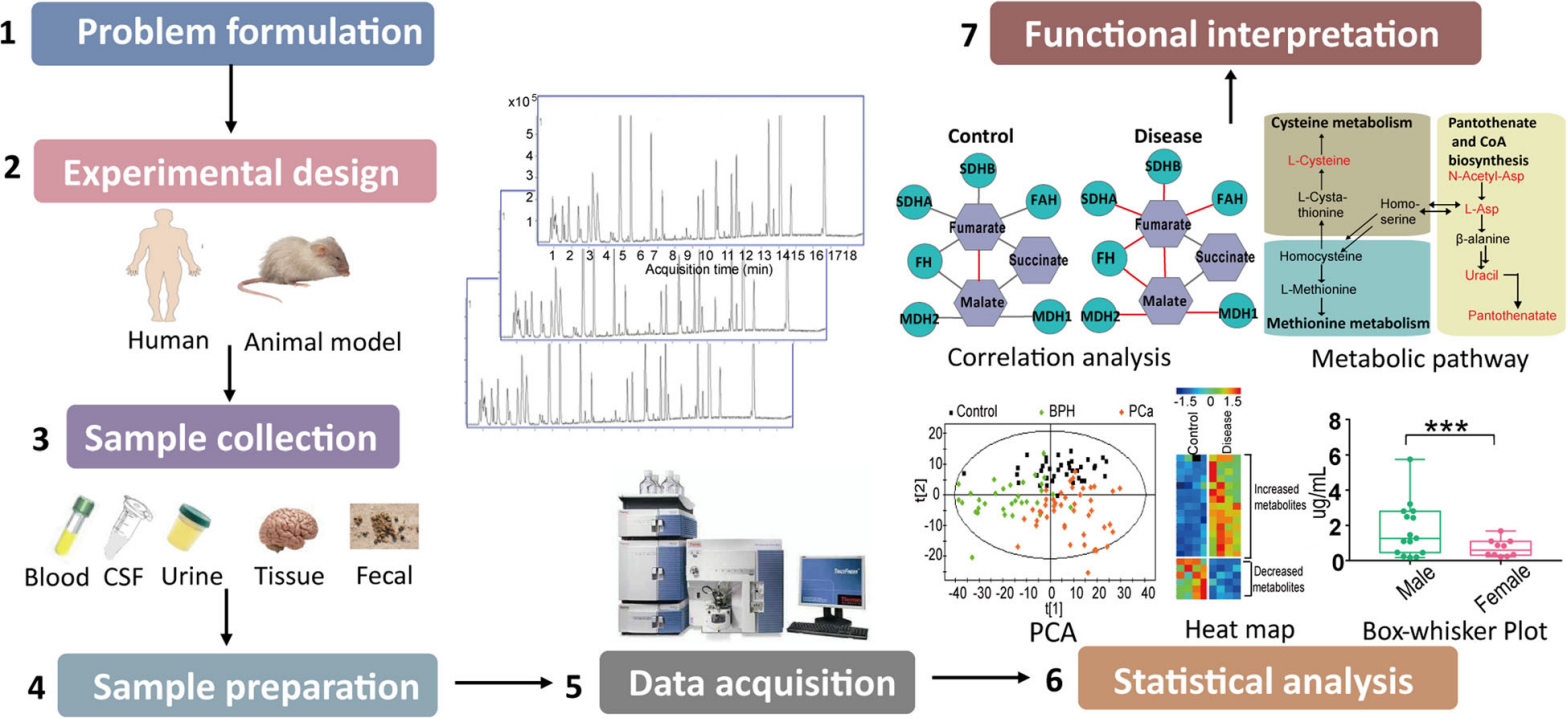
Analytical workflow of metabolomics studies. The typical metabolomics study including experimental design, sample collection, sample preparation, data acquisition, statistical analysis and functional interpretation stages

Metabolomics is an emerging technique that aims to investigate the global changes of numerous metabolites within a given sample, followed by deep data mining and bioinformatic analysis [16, 17] (Fig. 1). These metabolites are not only endogenous, but are also derived from the metabolism of pharmaceuticals, environmental chemicals, and the co-metabolism between host and gut microbiota [17]. Minor changes of endogenous and exogenous factors can be reflected at the level of metabolites; thus, the study of metabolomics has immense potential to link the genetic, environmental, and physiological elements to specific pathological states [18]. In the past decades, metabolomics has become a powerful tool for investigating metabolic processes, identifying potential biomarkers and unraveling metabolic reprogramming in various diseases [5, 19–21]. Advancements and achievements in both biological sample preparation and instrumental techniques have made the high-throughput analysis of a broad range of metabolites possible, stimulating a great interest regarding its potential application in PD research.

In this review, we summarized major improvements in analytical platforms and recent advancements in metabolomics studies, and discussed the advantage and limitation of each methodology. Then, we reviewed applications of metabolomics in PD research, and discussed the major metabolic findings in the metabolome of cerebrospinal fluid (CSF), blood, urine, feces and brain tissue in clinical populations as well as in experimental PD models. Finally, we outlined several abnormal metabolic pathways in PD, which may improve our knowledge on the molecular mechanisms mediating PD development, which can help develop new therapeutic strategies for this devastating disease.

### Major analytical platforms in metabolomics research

Nuclear magnetic resonance (NMR) spectroscopy and mass spectrometry (MS) are two predominant analytical platforms used in metabolomics [22]. Particularly, chromatograph-MS coupled systems including liquid chromatography-MS (LC-MS) and gas chromatography-MS (GC-MS) are the most popular techniques.

### Nuclear magnetic resonance spectroscopy

NMR is a powerful tool commonly used for the identification of metabolites. It offers various relevant and excellent attributes such as simple sample preparation, short analysis time, robust signal, and absolute quantification of metabolites [23]. However, the relative low sensitivity of NMR makes it incapable of measuring low-abundance metabolites. Due to the absence of a proper separation system, thousands of metabolites signals are overlapped, which make accurate structure identification a complicated and difficult task. Nonetheless, recent technological achievements have minimized these drawbacks and have improved the sensitivity and resolution of NMR techniques; the applications of highly sensitive cryoprobes and microprobes benefit to detect low-abundance metabolites with the detection limit reduced by 3 ~ 5 times [24, 25]. In addition, advanced NMR methods include two-dimensional (2D) NMR techniques, such as HSQC and TOCSY as well as hyphenated LC-MS-NMR, which have made great progress in recent years, improving both spectral resolution and metabolite identification capabilities [26, 27].

### Gas chromatography-mass spectrometry

GC-MS has been extensively used in metabolomics, particularly given its high separation power and reliable structure annotation capacity [28]. GC can be coupled to diverse types of mass analyzers, such as single quadrupole (Q), triple quadrupole (QqQ), ion trap (IT), and time of flight (TOF). Recently, the newly developed GC/Q-Orbitrap MS system has been shown to greatly improve the ability to identify unknown metabolites, due to its higher sensitivity, resolution, and mass accuracy [29]. In addition, chromatography separation techniques have been also improved. By combining two orthogonal columns, 2D GC yielded a multiplicative increase in peak capacity [30].

GC-MS is mainly used to analyze volatile (i.e., naturally volatile and made volatile by derivatization) and thermally stable metabolites. Among a multitude of chemical derivatization methods, a two-step process that includes oximation followed by trimethylsilylation, provides a broad coverage of metabolites and is currently the most commonly used approach [31]. The metabolites detected by GC-MS are mainly associated with tricarboxylic acid (TCA) cycle, glycolysis, urea cycle, amino acid metabolism, and fatty acid metabolism, among others. Recently, a group of fast and sensitive GC-MS-based methods have been developed for the quantification of short-chain and medium-chain fatty acids, and proved to be effective tools for exploring the effects of host-gut microbiota [32, 33]. GC-MS has been also used to explore the dysregulation of neurotransmitter, hormones, and purine metabolism in different neurological diseases [34, 35].

### Liquid chromatography-mass spectrometry

LC-MS is a widely used analytical platform in metabolomics research. Reverse-phase liquid chromatography (RPLC) and hydrophilic interaction liquid chromatography (HILIC) are two major chromatographic separation techniques [36], which provide complementary metabolic information [37]. Recently, 2D and multidimensional LC have emerged as powerful analytical techniques that provide higher peak capacity and better resolution by combining two or more columns with orthogonal characteristics [36]. The newly established 2D-LC-MS method enables the simultaneous analysis of metabolome and lipidome in one single run and is viewed as an efficient tool for large-scale metabolomics studies with a limited amount of samples [38].

Untargeted and targeted analyses are two traditional strategies for metabolomics studies [39]. Untargeted metabolomics has the best metabolites coverage, however it holds poor reproducibility and limited sensitivity for low-abundance metabolites [40]. Targeted metabolomics has been regarded as the gold standard for metabolite quantification due to its high sensitivity, broad dynamic range, and reliable quantification accuracy, although it covers limited pre-known metabolic information [41]. Dynamic multiple reaction monitoring (MRM)-based pseudo-targeted metabolomics quantification and parallel reaction monitoring (PRM)-based larger-scale targeted metabolomics quantification, are two newly emerged strategies, both of which can measure a large number of metabolites with reliable quantitative arrays and are now proved to be powerful tools for metabolomics studies [42].

Although all of these techniques enable simultaneous identification and quantitation of multitudinous metabolites coexisting in one single sample, none of them are able to cover the entire metabolome yet. Nevertheless, the combination of multiple analytical platforms can contribute to an improved metabolic coverage.

### Metabolomics studies in patients with PD

Initially, traditional targeted approaches were mainly implemented in the evaluation of a few selected metabolites of interest, including catecholamines, amino acids, purines and urate [43–45]. Until the last decade, untargeted metabolomics has been applied to PD research, counting on its enormous potential for the identification of novel biomarkers. Most of these studies are based on CSF and blood analysis, although some studies have examined other biological samples such as urine, feces or brain tissue. In the following section, we will review primary metabolomics-based findings in the metabolome of different sample matrices obtained from PD patients.

### Cerebrospinal fluid metabolome

CSF composition abnormalities are directly related to pathological changes in the brain, making CSF one of the preferred samples for neuropathological studies. Given the marked depletion of the nigrostriatal dopaminergic neurotransmission in PD patients, measurements of dopamine levels and its metabolites may provide a path to the discovery of a reliable trait biomarker [43]. Using the LC- electrochemistry array (LCECA) based targeted approach, significant reduction of catecholamines including homovanillic acid [46] (HVA), dihydroxyphenylacetic acid (DOPAC), L-dopa, and dihydroxyphenylglycol has been reported in PD [43]. Among others, DOPAC levels showed high accuracy in distinguishing PD (including early onset) from controls. However, low levels of DOPAC are not specific for PD, and a marked reduction of catechols has been also observed in patients with other synucleinopathies like pure autonomic failure and multiple system atrophy (MSA) [43].

Other metabolites of interest in the CSF of PD are purines. An exploratory study investigating the levels of xanthine and HVA in PD versus controls using the LCECA platform, found that the ratio of xanthine to HVA in CSF permitted an excellent distinction of PD from controls [47]. In addition, statistically significant high-levels of 8-hydroxy-2-deoxyguanosine (8-OHdG) and 8-hydroxyguanosine (8-OHG) were observed in PD, when compared to controls in two independent studies [48, 49], indicating that oxidative stress markers could be potentially useful in the diagnosis of PD.

The wide metabolites coverage and high-throughput analysis of untargeted metabolomics make it an effective tool for the discovery of novel PD biomarkers. Using GC-TOF/MS-based metabolomics, significant reductions in tryptophan, creatinine, and 3-hydroxyisovalerate levels were reported in PD compared to controls [50]. Another study based on NMR metabolomics further identified a panel of metabolites (alanine, creatinine, dimethylamine, glucose, lactate, mannose, phenylalanine, 3-hydroxyisobutyric acid and 3-hydroxyisovaleric acid) that exhibited a good capacity to discriminate PD from controls [51]. Recently, using untargeted MS-driven approach, specific metabolic signatures of PD in early stages of the disease were uncovered [5, 52]. These PD-specific metabolites have been shown to be involved in antioxidative stress responses, and metabolic pathways of sphingolipid, glycerophospholipid and amino acid, which may aid in the accurate diagnosis of early-stage PD [5, 52]. It was noteworthy that Stoessel et al. demonstrated a relatively high overlapping of metabolome in CSF and blood, implying a joint analysis of multiple biofluids collected from the same subject will be more valuable in reflecting the overall metabolism [52].

### Blood metabolome

Compared with CSF metabolomics research, a larger number of untargeted metabolomics-based studies have been reported using plasma/serum samples, possibly due to its minimally invasive nature and relatively easy availability of blood samples. We summarized the major findings of blood metabolome studies of PD published over the past decade (Table 1). In general, case-control studies accounted for the majority, except for several studies including subgroups of PD such as *LRRK2* mutation [16] and patients with or without LID [6]. The differential metabolites between PD and matched controls can be classified as amino acids, fatty acids, acylcarnitines, lipids, purines, organic acids and sugars, which are parts of branched chain amino acids (BCAAs) metabolism, tryptophan metabolism, lipid metabolism, energy metabolism, purine metabolism, and oxidative stress/redox homeostasis pathways. Recently, a group of studies consistently demonstrated the dysregulation of kynurenine pathway in PD [19, 53, 54]. The alterations of kynurenine metabolites in PD not only provide potential biomarker candidates and novel avenues for investigating PD pathogenesis, but also offer a new therapeutic strategy for PD with the supplement of kynurenic acid or the reduction of quinolinic acid using kynurenine 3-monooxygenase inhibitors [19].

**Table 1:** Overview of metabolomic studies in the blood metabolome of PD clinical populations

Analytical platform	Subjects	Differential metabolites/metabolic pathways	Statistics	Validation	Reference
HILIC-TOF/MS	Early PD (*n* = 80) Controls (*n* = 76)	Ethanolamine, N-Lauroylglycine, Alpha-N-Phenylacetyl-L-glutamine, Sarcosine, Glu-Ile, 1,3-Dimethyluracil, Arg-Ala, PCs, SMs, Lyso-PAF C-16, etc.	ROC (AUC = 0.80)	No	Stoessel D et al. [52]
CE-TOF/MS LC-TOOF/MS	PD (*n* = 109) Controls (*n* = 32)	Long chain acylcarnitines	ROC (AUC = 0.895)	Yes	Saiki S et al. [102]
LC-MS	PD (LID, *n* = 10, non-LID, *n* = 8, unmedicated, *n* = 8) Controls (*n* = 14)	3-hydroxykynurenine/kynurenic acid Ratio	t-test, *p* < 0.05	No	Havelund JF et al. [6]
Nontargeted MS-based metabolomics	Early PD (*n* = 41) Controls (*n* = 40)	Hexanoylglutamine, Decanoylcarnitine, Myristoleoylcarnitine, Octanoylcarnitine, Oleoylcarnitine, Palmitoleoylcarnitine, Suberoylcarnitine, Octadecanedioate, 3-hydroxysebacate	ROC (AUC = 0.857)	No	Burté F et al. [111]
UPLC-MS/MS GC-MS	PD (*n* = 35) Controls (*n* = 15)	Lower levels of tryptophan, caffeine, bilirubin and ergothioneine; higher levels of levodopa metabolites and biliverdin	random forest classification	No	Hatano T et al. [112]
NMR	PD (*n* = 43) Controls (*n* = 37)	Myoinositol, sorbitol, citrate, acetate, succinate and pyruvate	PLS-DA	No	Ahmed SS et al. [113]
LCECA	LRRK2 PD (*n* = 12) idiopathic PD (*n* = 41) Controls (*n* = 46)	Purine metabolism (uric acid, hypoxanthine, xanthine, etc.)	PLS-DA	No	Johansen KK et al. [16]
LCECA	PD (*n* = 66) Controls (*n* = 25)	8-OHdG, glutathione, uric acid	PLS-DA	No	Bogdanov M et al. [17]
GC-TOFMS	PD (*n* = 20) Controls (*n* = 20)	Amino acids (pyroglutamate and 2-oxoisocaproate), C16-C18 saturated and unsaturated fatty acids	OPLS-DA	No	Trupp M et al. [50]
LC-MS GC-MS	PD (*n* = 82) RLS (*n* = 95) Controls (*n* = 1272)	Long-chain (polyunsaturated) fatty acids, inositol metabolites		No	Kassubek J et al. [101]
UPLC-TOF/MS	PD (cohort 1, *n* = 82, cohort 2, *n* = 118) Controls (cohort 1, *n* = 82, cohort 2, *n* = 37) Huntington’s disease (cohort2, *n* = 22)	Kynurenic acid, quinolinic acid, ratio of kynurenic acid /kynurenine, ratio of quinolinic acid/ kynurenic acid	OPLS-DA	Yes	Chang KH et al. [19]
LC-QE/MS	Slow. PD (*n* = 41) Rapid. PD (*n* = 39) Controls (*n* = 20)	N8-acetyl spermidine	OPLS-DA	No	Roede JR et al. [49]

Plenty of biological, epidemiological and clinical studies have convergently suggested urate as a promising biomarker of the risk, diagnosis and prognosis of PD. Significantly reduced level of urate in both CSF and blood of PD was reported compared to controls [45, 55], and a high level of urate may indicate a lower risk, slower progress of the disease [55–57]. As an important endogenous antioxidant [55], increased level of urate may contribute to fight against oxidative stress in the pathogenesis of PD [58]. Detailed elaborations of the correlation between urate and PD have been reviewed elsewhere [55, 59].

As previously mentioned, PD is a multifactorial disease with compelling epidemiological data that suggest a probable link between traumatic brain injury (TBI) and PD; however, such association is still controversial due to the lack of mechanistic basis [60]. Based on untargeted and targeted LC-MS approaches, a statistically significant alteration of glutamate level was identified in blood samples from both TBI and PD, implying a possible “excitotoxic” link between TBI and PD [61]. Additionally, the overlap of clinical symptoms between PD and other neurodegenerative diseases, such as primary progressive multiple sclerosis (PPMS), progressive supranuclear palsy (PAP) and MSA often lead to high rates of misdiagnosis for PD [3]. Recently, two studies using NMR and LC-MS based metabolomics profiled the blood metabolome of patients with PD, PPMS, PAP, and MSA versus controls, showing that BCAAs were significantly increased in PD, PAP, and MSA compared to controls [3], and a set of 20 metabolites involved in glycerophospholipid and linoleic acid pathways were specifically altered in PPMS which were distinguishable from PD [48].

Metabolomics can also reveal biomolecular and pathway changes implicated in disease onset and progression. To this end, it has been reported that the level of N8-acetyl spermidine may be a predictive marker for a fast motor progression disease phenotype, which may provide a novel strategy for delaying or slowing down the progression of PD [49]. Based on metabolomics approaches, plasma metabolic profiles of serine, purine, fatty acid, polyamines, and metabolites associated with tryptophan metabolism have presented a high correlation with the progression of PD [6, 19, 53]. Additionally, it has been shown that kynurenine metabolism is also associated with the development of LID, and increased plasma ratio of 3-hydroxykynurenine /kynurenic acid may predict the possibility of LID [6, 19].

### Urine metabolome

Given the easy availability and noninvasive sampling of urine samples, they are ideal sources of biomarkers for clinical analysis. Incipiently, research studies were focused on evaluating oxidative stress markers by targeted analysis strategies [62–64]. Under the attack of reactive oxidative species, the bases in DNA can be hydroxylated and oxidized; 8-OHdG and 8-OHG are two of the most prominent products of DNA impairment [65]. The resulting 8-OHdG can be excreted into urine without further metabolism, which is regarded as an indicator of oxidative DNA damage [63]. Previous studies demonstrated elevation of 8-OHdG level in the substantia nigra of the brain [66, 67] as well as in the serum and CSF of PD [68]. Based on targeted analysis, it was demonstrated that level of 8-OHdG in the urine alone or the ratio of 8-OHdG/2′-deoxyguanosine can significantly distinguish PD from the controls [63]. In addition, level of urinary 8-OHdG showed a progressive increase with PD advances, suggesting that it may be an useful biomarker to track disease progression [62]. Moreover, utilizing non-targeted metabolomic profiling method, biopyrrin was identified as a new marker for sporadic PD [69]. Biopyrrin, the oxidative product of bilirubin, has been regarded as the indicator of increased oxidative stress, showed high predictable ability for different stages of PD (AUC = 0.95 ~ 0.98) [69].

The urine, which contains abundant metabolites, has seldom been investigated by untargeted metabolomics in PD research. Using LC-MS and a random forest model, a recent study profiled urinary metabolites in sporadic PD versus controls and identified a panel of metabolites that yielded with > 90% accuracy in distinguishing PD from controls [70]. Based on GC-MS and LC-MS technologies, another study of the same group identified 18 metabolites that showed progressive increases with the development of PD [71]. Both of these studies indicate that the dysregulation of steroidogenesis, glycine derivation, tryptophan and phenylalanine metabolic pathways are related to the development and progression of PD [71]. Recently, an assay combining UPLC-MS/MS with in situ selective derivatization was developed to detect a wide range of neurochemicals in urine samples, presenting a promising analytical platform to screen potential biomarkers that can aid in the diagnostic accuracy and tracking of PD prognosis [72].

### Fecal metabolome

Recent investigations have highlighted the crucial role of the gut microbiota in the development of neurodegenerative diseases including PD [73–75]. Fecal metabolome, can provide information regarding the metabolic interactions between host, diet, and gut microbes, presenting a promising avenue to “fingerprint” the functional status of the intestinal microbiota and explore links between microbiome and host phenotypes [76]. Fecal metabolomics has been widely used in both biomarker identification and functional annotation for various diseases, such as irritable bowel syndrome, nonalcoholic fatty liver disease, obesity, and autism [77–79]; however, this approach has been rarely used for the investigation of neurodegenerative diseases. Recently, a reduction of fecal short chain fatty acids (SCFAs) was identified in PD, when compared to controls by GC-based quantitative analysis [80]. Since SCFAs can regulate the function of the enteric nervous system and promote gastrointestinal motility, a reduction of SCFAs might contribute to the development of gastrointestinal motility disorders in PD [80, 81].

### Tissue metabolome

Human brain metabolomics studies are mainly based on NMR spectroscopy techniques, which enable non-destructive detection of the chemical composition of a specific area in a living body. In vivo PD brain metabolomics based on NMR spectroscopy has been reviewed elsewhere [82]. In summary, these studies are mainly focused on the mitochondrial dysfunctions observed in PD patients by tracing the levels of creatine, phosphocreatine, ATP, high-energy phosphates, phospholipids, and lactate [82–85], and indicate impaired mitochondrial oxidative phosphorylation events in the brain of PD patients, even in the absence of a clinical phenotype. Moreover, the mitochondrial machinery in patients carrying a *PINK1* mutation, was more susceptible to these events than idiopathic PD [86]. Notably, the combined evaluation of N-acetylaspartate/creatine levels from both the pontine base and putamen in brain tissues may offer effective strategies to distinguish MSA with predominant Parkinsonism from PD, as reported by a number of studies [82, 87, 88]. By implementing a LC-MS-based lipidomics technique, a recent study identified abnormal levels of diacylglycerols in the frontal cortex of PD patients who presented no neocortical pathology [89]. These data suggest that the elevation of plasma levels of diacylglycerols in PD may be a promising marker for neurodegenerative processes that ought to be further investigated.

### Metabolomics studies in PD models

Although various types of animal models have been established for PD research, only a few of them have been used for metabolomics studies. We summarized these studies in Table 2, the genetic models used include α-synuclein (α-Syn) knockout, α-Syn transgenic, α-Syn overexpressed [90–92] and Park2 knockout animal models [93], while toxicological models are mainly induced by paraquat, rotenone, 1-methyl-4-phenyl-1,2,3,6-tetrahydropyridine (MPTP), methyl-4-phenylpyridinium, and 6-hydroxydopamine (6-OHDA) [94–96]. In these animal studies, the metabolic profiles identified originated primarily from brain tissues (whole brain or specific areas), which better reflect the patho-physiological changes.

**Table 2:** Overview of metabolomic studies in experimental models of PD

Analytical platform	Models	Differential metabolites/metabolic pathways	Reference
GC-MS	PQ-exposed Drosophila	Amino acids, fatty acids, carbohydrates, etc.	Shukla AK et al. [21]
NMR, DI-ESI-MS	PQ-exposed dopaminergic cell	Pentose phosphate pathway (PPP), glycolysis, TCA cycle	Lei S et al. [98]
NMR	MPTP-induced PD goldfish	BCAAs, alanine, myo-inositol, fatty acids, taurine, creatinine, N-acetylaspartate, (phospho)creatine, phosphatidylcholines, cholesterols,	Lu Z et al. [114]
LC-MS	Rotenone-treated rats	Oxidizable PUFA-containing cardiolipin	Tyurina YY et al. [99]
HPLC-ESI-MS/MS	6-OHDA-induced rats	Phosphatidylcholine and lysophosphotidylcholine lipid	Farmer K et al. [100]
MS-based lipidomics	α-Syn KO, α-Syn TG mice	Age-related phospholipids	Rappley I et al. [90]
NMR, LC-MS	Mouse model of prodromal PD	Taurine and hypotaurine metabolism, bile acid biosynthesis, glycine, serine, and threonine metabolism, and citric acid cycle	Graham SF et al. [97]
NMR, DIESI-MS	PQ -induced dopaminergic N27 cells	Glucose metabolism	Anandhan A et al. [92]
NMR	6-OHDA-induced rats	GABA, Glu, Gln, lactate, N-acetylaspartate, creatine, taurine, and myo-inositol.	Zheng H et al. [115]
UPLC-QTOF-MS	MPTP-induced PD mice	Tyrosine metabolism, mitochondrial beta-oxidation of long chain saturated fatty acids, fatty acid metabolism, methionine metabolism, and sphingolipid metabolism	Li XZ et al. [116]
UPLC-MS	α-Syn A53T TG mice	Alanine metabolism, redox and acetyl-CoA biosynthesis pathways	Chen X et la. [91]
LC-MS	Park2 kO mice, CCCP-treated mice	Energy metabolism	Poliquin PO et al. [93]

Recently, a mouse model of prodromal PD was established via unilateral injection of preformed α-Syn fibrils in the olfactory bulb [97]. Contrary to earlier reports, both brain tissue and serum were collected and subjected to metabolomics analysis for the development of early diagnostic markers of PD. The pathway enrichment of the brain data suggested a dysregulation of taurine and hypotaurine metabolism, bile acid biosynthesis, glycine, serine and threonine metabolism and the TCA cycle were in correlation with the onset and progression of α-Syn pathology, while the results from the serum highlighted only phospholipid metabolism alterations in α-Syn PFF-injected mice that may provide evidence for the possible interaction between lipid metabolism and α-Syn aggregation [97].

Consistently, studies from three different groups, although, based on different animal models, showed a relatively limited impact of the genotype on metabolites levels, when compared to aging or toxic exposure [90, 91, 93]. It was found that lipidomic profiles were age-dependent in the wild type-mice, and the α-Syn genotype-dependent phospholipid differences indicated a strong interaction of age and α-Syn gene dosage [90]. Based on metabolomics and mathematical model, Poliquin et al. investigated and compared the energy deregulation in the cerebral tissue of genetic (Park2 knockout) and CCCP-induced models of PD, and the findings suggested that genetic perturbations are not sufficient to lead to significant metabolic changes compared to toxin exposure [93].

Toxicological models can, to some extent, simulate the roles of oxidative stress, mitochondrial dysfunction, and dopamine metabolism associated with the pathogenesis of PD, which may contribute to α-Syn misfolding and aggregation [98]. A generally profound reduction of lipid species was found in brain tissues of PD models induced by rotenone and 6-OHDA, except for a few lipids that showed elevated levels, such as mono-oxygenated cardiolipins (CLs) [99] and several lysophosphatidylcholines [100], all pointing to an increased oxidative damage, insufficient energy and mitochondrial dysfunction in PD. In contrast, the most striking metabolic alterations induced by paraquat treatment were the selective up-regulation of the pentose phosphate pathway (PPP) and the down-regulation of the glycolysis and the TCA cycle [92, 98]. Powers et al. indicated that the alterations in energy metabolism were not bystanders to energy failure, but also played important roles in dopaminergic cell death via gene (α-Syn)-environment (paraquat) interactions [92].

Metabolic alteration differences among the various genetic/toxic-induced models highlight the multifactorial nature of PD. Future longitudinal metabolic profiling studies based on representative animal models will be able to contribute to a better understanding of the onset and development of the disease.

### Dysregulation of metabolic pathways in PD

**Fig. 2:**
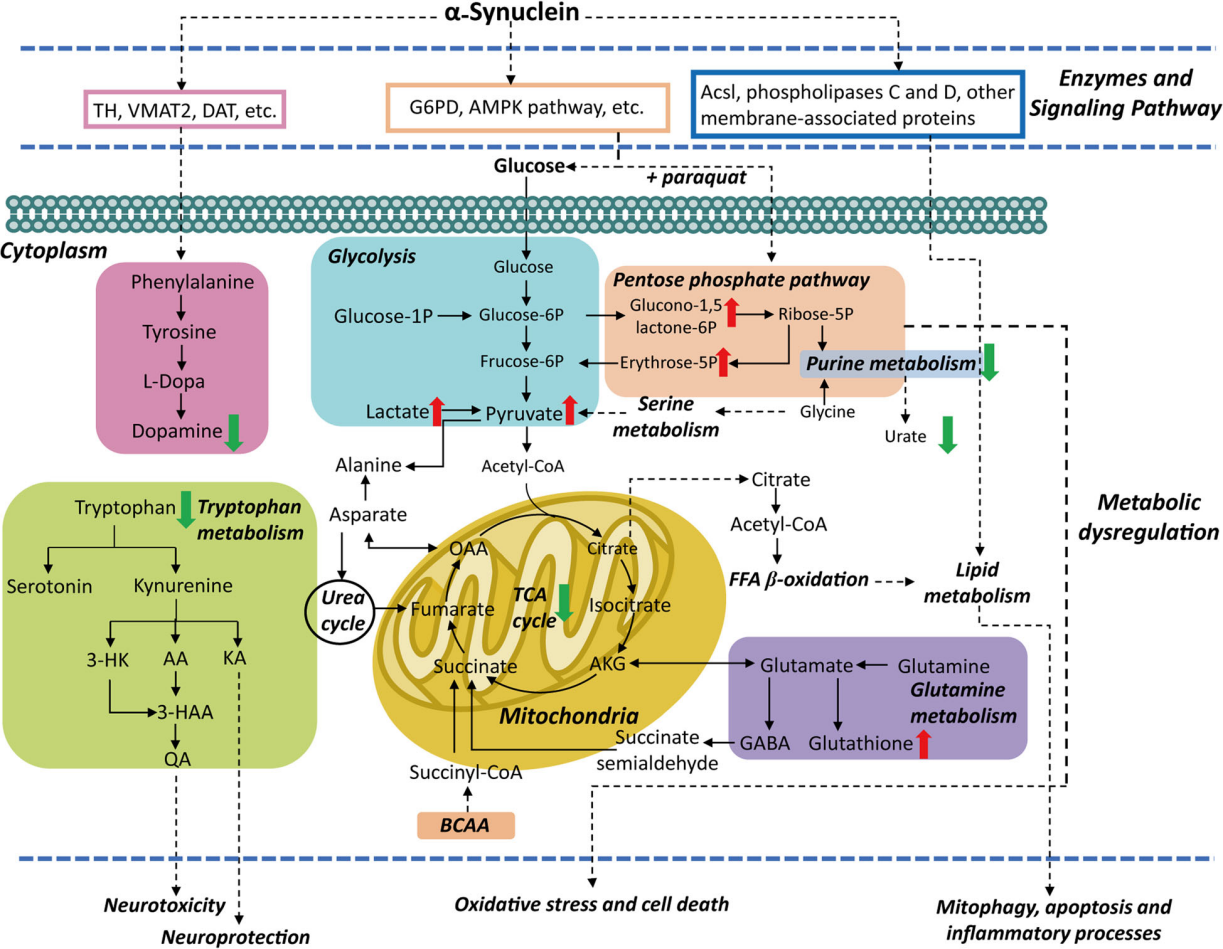
Overview of the metabolic pathway dysregulations in PD. The alterations of some metabolites may be different (upregulation or downregulation) in different sample matrices of drug-naïve patients, L-dopa treated patients or different PD models, thus the changes of these metabolites are not shown

PD exhibits high heterogeneity, having multiple pathways and molecular mechanisms mediating its molecular pathogenesis. Based on metabolomic findings in clinical and experimental models, the metabolic pathways that are majorly perturbed in PD are related to the metabolism of lipids, energy (TCA cycle, glycolysis, PPP, BCAA, acylcarnitines), fatty acids, bile acids, polyamine, and amino acids (Fig. 2) [49, 50, 90, 98, 99, 101, 102].

Markedly, a significant reduction of catecholamine metabolite level has been shown for both PD patients and PD animal models, due to the marked depletion of nigrostriatal dopaminergic neurons in PD pathology. The treatment with the different dopaminergic drugs available could selectively increase the levels of these metabolites [103]. In addition, L-DOPA treatment has been also shown to have a profound impact on aromatic amino acid metabolic pathways. Notably, kynurenine metabolism, a pathway of tryptophan metabolism, may have a strong link with PD progression and the risk of LID development [6, 53].

Moreover, accumulating evidence have corroborated that α-Syn plays an important role in the pathogenesis of PD via lipid binding, regulating the composition of membrane, modulating fatty acid metabolism and influencing the release of the neurotransmitter by interacting with specific lipids [90, 104]. The general reduction of lipids levels, such as polyunsaturated fatty acids (PUFAs) and phospholipids in PD models, presumably due to an excess of oxidative stress, given that membrane phospholipids are major targets for free radicals. The alterations of PUFA-CLs and oxidized CLs not only point to mitochondrial dysfunction, but also indicate possible mitophagy and apoptosis processes in the development of PD [99].

In normal brain function, threonine and glycine can be converted into creatine, which in turn provides phosphate groups for ADP to produce ATP [97]. TCA cycle is an important pathway in the production of ATP through the oxidative phosphorylation of acetyl-CoA in the mitochondrial. With the beginning of α-Syn aggregation during the onset of the neurodegenerative processes in PD, the metabolism of glycine, serine, and threonine, as well as the TCA cycle, appear to be downregulated [97], which indicate an energy insufficient and mitochondrial dysfunction in PD. In paraquat-induced models, central carbon metabolism has been shown to contribute to dopaminergic cell death by regulating the effect of α-Syn on paraquat toxicity, and inhibiting the metabolism and transport of glucose and PPP can reduce paraquat-induced oxidative stress and cell death [92, 98, 105].

Furthermore, alteration of bile acids has been found in both PD patients and PD animal models [97, 102]. Bile acids are produced in the liver from cholesterol and then metabolized by gut microbiota-derived enzymes into secondary bile acids such as ursodeoxycholic acid or tauroursodeoxycholic acid [106]. In addition, it has been demonstrated that tauroursodeoxycholic acid can rescue mitochondrial function and prevent MPTP-induced dopaminergic cell death in different animal models of PD [107].

Currently, the drugs designed to treat or prevent PD are focused on the prevention or elimination of α-Syn aggregation; however, no successful cases have been reported yet. In contrast, an alternative and more effective strategy may be the development of specific inhibitors/activators designed to directly target metabolic processes [108, 109]. Importantly, metabolomics studies can provide comprehensive biochemical underpinnings to unravel the molecular mechanisms of PD pathogenesis, offering biomarkers that reflect pathological processes and may substantially improve drug development strategies against PD.

## Conclusions

### Merits and caveats of metabolomics for PD research

Metabolic changes are the direct results of alterations in protein and enzyme activities. Therefore, metabolomics may offer valuable information on PD-related physiological process, molecular interactions and metabolic pathways. By providing an overall “fingerprint” of metabolite alterations in multiple biofluids and tissues, metabolomics has provided a myriad of potential biomarkers and therapeutic targets. Nevertheless, metabolomics is still in its infancy, particularly when it comes to PD research. The identification of the unknown metabolites is one of the major challenges. Although great progress has been made during the last decade, the public and commercial databases of metabolites are still limited and incomplete, the current metabolic findings may be only the “tip of the iceberg” of the whole picture of PD etiology. Another important issue is the heterogeneous nature of the individuals. Differences in genotype, medical history, disease progression, lifestyle and diet, etc. of the subjects are likely to affect their metabolome, which may obscure the direct influence from the disease. Besides, the reported works usually used different analytical techniques and different sample preparation methods based on different designs, thus it is not surprised to obtain controversial conclusions.

### Future perspectives

Confirmatory studies based on optimized experimental protocols are urgently needed. The potential biomarkers and metabolic pathways revealed in the present studies require to be validated by independent large-scale populations. As highlighted above, further stratification of PD may allow the identification of specific targets among the different subtypes of PD. Also, a joint analysis of multiple biofluids and tissues using complementary analytical platforms should be employed in parallel to reveal the “bigger picture” for an in-depth biological investigation. It is noteworthy that other related diseases that have similar clinical symptoms with PD should be included in future studies. Identifying metabolites that are specifically changed in PD compared with controls and other related diseases will be of great significance for clinical differential diagnosis. In addition, accumulating evidences suggest that microbiome dysbiosis and changes in microbial metabolite levels are strongly associated with the pathogenesis of PD [74, 75]. Several metabolites involved in the regulation of brain function have been found in the gut, the concentrations of which can be regulated by gut microbiota, further influencing the function of neurons [110]. Given that metabolomics has been shown to be a powerful tool to fingerprint metabolic profiles in multiple matrices, the combination of metabolomics with other techniques, such as metagenomics, proteomics, and transcriptomics may lead to a better understanding of host–microbe interactions and yield potential novel biomarkers for PD diagnosis and therapeutic targets for effective treatment options.

## Abbreviations

6-OHDA: 6-hydroxydopamine
8-OHdG: 8-hydroxy-2-deoxyguanosine
8-OHG: 8-hydroxyguanosine
AUC: Area under the curve
BCAA: Branched chain amino acid
CE: Capillary electrophoresis
CL: Cardiolipin
CSF: Cerebrospinal fluid
DOPAC: Dihydroxyphenylacetic acid
ECA: Electrochemical detection
GC: Gas chromatography
HVA: Homovanillic acid
IT: Ion trap
LC: Liquid chromatography
LID: L-dopa induced dyskinesia
MPTP: 1-methyl-4-phenyl-1,2,3,6-tetrahydropyridine
MS: Mass spectrometry
MSA: Multiple system atrophy
NMR: Nuclear magnetic resonance
PAP: Progressive supranuclear palsy
PD: Parkinson’s disease
PLS-DA: partial least squares-discriminate analysis
PPMS: Primary progressive multiple sclerosis
Q: Quadrupole
QqQ: Triple quadrupole
RPLC: Reverse-phase liquid chromatography HILIC Hydrophilic interaction liquid chromatography
SCFAs: Short chain fatty acids
TBI: Traumatic brain injury
TCA: Tricarboxylic acid
TOF: Time of flight
α-Syn: α-synuclein
